# Heterologous Expression of a Potential ‘*Paulownia fortunei*’ MYB Factor Gene, *PfMYB90*, Improves Salt and Cold Tolerance in *Arabidopsis*

**DOI:** 10.3390/plants14010024

**Published:** 2024-12-25

**Authors:** Hongling Wang, Shizheng Shi, Guijie Luo, Ruifang Huang, Dezong Sui, Yunpeng Gao, Lei Wang

**Affiliations:** 1 Jiangsu Academy of Forestry, Nanjing 211153, China; 2 Suqian Institute of Agricultural Sciences, Jiangsu Academy of Agricultural Sciences, Suqian 223800, China

**Keywords:** *Paulownia fortunei*, *PfMYB90*, salt stress, cold stress

## Abstract

The paulownia tree belongs to the Paulowniaceae family. Paulownia has strong vitality; has strong adaptability to harsh environmental conditions; and can be used as building raw material, as well as processing drugs and having other purposes. In the research field of MYB transcription factors of the paulownia tree, it is rare to discuss the resistance to abiotic stress. The research in this area has not received sufficient attention and depth, which also indicates an important potential direction for future research. In this study, we performed bioinformatics analysis of the stress-related gene PfMYB90, a potential transcription factor, and investigated its mechanism of action under salt and cold stresses. *PfMYB90* was strongly expressed in the fully unfolded leaf and root of plants in both stress treatments. Transgenic *PfMYB90 Arabidopsis* plants had a greater survival rate under salt and cold stresses, and the degree of leaf damage was comparatively smaller, according to phenotypic observation and survival rate calculations. By measuring the corresponding physiological indexes after stress and detecting the expression levels of corresponding stress genes (*AtNHX1*, *AtSOS1*, *AtSOS2*, *AtSOS3*, *AtCBF1*, *AtCBF3*, *AtCOR15a*, *AtRD29a*), it was found that after *PfMYB90* gene transfer, *Arabidopsis* showed strong tolerance to salt and cold stresses. This is consistent with the results mentioned above. This transgenic technology enables *Arabidopsis* to survive under adverse environmental conditions, allowing it to maintain a relatively stable growth state despite salt accumulation and cold stress. Therefore, *PfMYB90* may be a key gene in the regulatory network of salt damage and cold damage, as well as one of the key transcription factors for *Paulownia fortunei* environmental conditions.

## 1. Introduction

The Earth’s environment is in the process of constant change, and in order to continue life in such changes, some plants have evolved matching adaptability, which makes some plants can survive even in an extremely unfavorable environment for growth and development. This phenomenon brought by the environment to pressure plant life activities is also known as ‘abiotic stress’ [1]. In recent years, a series of studies on stress-related transcription factors have been carried out to reveal the root cause of plant ‘stress resistance’ through molecular means, which is of great significance for maintaining ecological balance, improving the quality of agricultural products and ensuring national food sovereignty [2,3,4].

The R2R3-MYB subgroup is the largest subgroup in MYB transcription factors (TFs), and the structure of this subgroup consists of two parts: the DNA-binding domain at the N-terminal and the conserved amino acid sequence motif domain at the C-terminal [5]. The MYB domain consists of four incomplete repeat sequences (R1, R2, R3, and R4), forming a helix–helix structure [6,7] This subgroup of transcription factors plays a significant role in response to stress regulation and resistance to biological and abiotic stresses [8,9].

So far, researchers have found a large number of R2R3-MYB TFs from many different plants. *XsMYB30*, an R2R3-MYB gene family member, was found in *Xanthoceras sorbifolium*. *XsMYB30* was induced by drought, salt, ABA MeJA, and SA [10]. *OsMYB91* can coordinate the life activities of rice in saline-alkali soil and ensure the normal growth of rice. Salt stress induces rapid changes in DNA methylation and histone modification patterns of *OsMYB91* promoter and gene transcription region [11]. *GhMYB73* is a member of the R2R3-MYB family isolated from *Gossypium hirsutum*. When this gene was overexpressed, transgenic *Arabidopsis thaliana* was more resilient to salt stress, while cotton increased sensitivity to salt stress after silencing this gene, which reversed verified the regulatory role of *GhMYB73* on salt stress [12]. Previously, the main research direction of MYB TFs in Paulownia was mainly on its effects on disease resistance [13,14], secondary metabolism [15], and morphological characteristics [16]. There is little research on abiotic stress.

The *Paulownia fortunei* belongs to the genus Paulownia in the Scrophulariaceae. It is a deciduous tree, a total of nine species and two varieties in China, cultivated or wild in the north of the three eastern provinces, Inner Mongolia, northern Xinjiang, Tibet, and other areas, and some areas are gradually introduced [17]. The dried paulownia tree is an excellent wood and is widely used in the manufacture of ships, furniture, and buildings [18]. The bark, leaves, and petals of paulownia tree can also be used as medicine, and the fruit of the paulownia tree has certain curative effects on asthma [19]. In some areas of northeast China, soil salinization and cold damage have hindered the planting of paulownia trees. Therefore, it is necessary to study the abiotic stress of paulownia trees. In this study, *PfMYB90*, a potential transcription factor highly related to cold resistance and saline-alkali resistance, was screened from the paulownia genome, with the intention of researching the gene function of the MYB TF family and offer theoretical backing for paulownia tree resistance research.

## 2. Results

### 2.1. Isolation, Identification, and Bioinformatics Analysis of PfMYB90

The *PfMYB90* (*Paulownia fortunei*) gene fragment was obtained by cloning based on the PCR technique. The CDS of *PfMYB90* contains a total of 906 nucleotide bases, encoding 301 amino acids, with Ser (9.0%), Lys (8.6%), and Asn (8.3%) accounting for the largest proportion (Figure S1). The predicted data showed that PfMYB90 had an average hydrophilic coefficient of −0.96, indicating that PfMYB90 is highly hydrophilic and can therefore be classified as a hydrophilic protein. The protein secondary structure of PfMYB90 was predicted and analyzed, and it was found that it consisted of 28.91% alpha-helix, 6.31% beta turn, 6.31% extended strand, and 58.47% random coil (Figure S2A). PfMYB90 contains two SANT conserved domains, 9–59 aa and 62–110 aa (Figure S2B). The PfMYB90 tertiary protein structure prediction results demonstrated that the tertiary structure prediction model was in agreement with the secondary protein structure and conserved domain predictions (Figure S2C).

**Figure 1:**
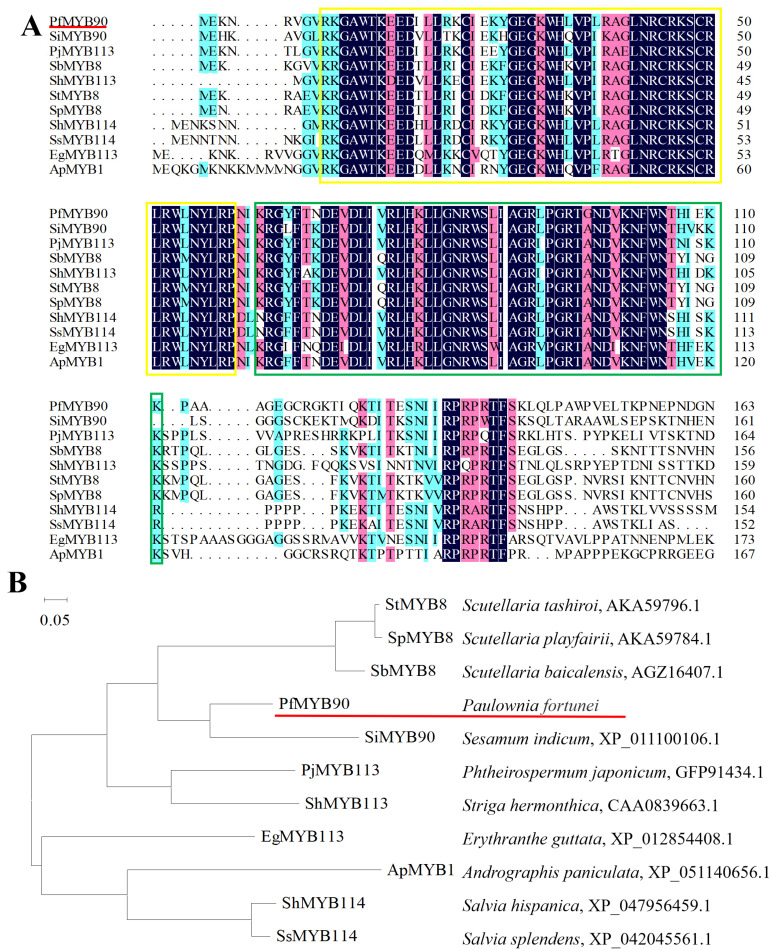
PfMYB90 protein multiple sequencing analysis. (**A**) Alignment of MYB sequence. (**B**) The evolution tree. Target proteins are represented by the red lines, while R-repeat structures are shown by the yellow and green boxes.

Multiple sequence alignment showed that PfMYB90, like other MYB proteins, contains R2 and R3 domains (Figure 1A). The phylogenetic tree analysis showed that SiMYB90 was the closest evolutionary relation to PfMYB90, followed by StMYB8, SbMYB8, and SpMYB8, which together formed the first cluster of the evolutionary tree (Figure 1B).

### 2.2. Subcellular Localization of PfMYB90 Protein

**Figure 2:**
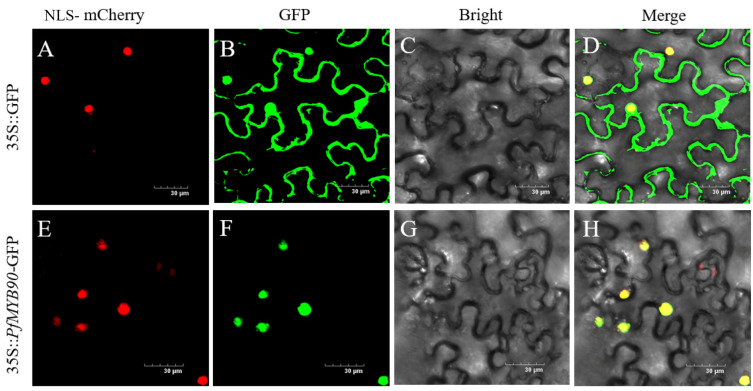
Subcellular location of PfMYB90. There are four frames in the image: (**A**,**E**) NLS-mCherry, (**B**,**F**) GFP, (**C**,**G**) Bright, and (**D**,**H**) Merge. bar = 30 μm.

The fusion vector *PfMYB90*-pCAMBIA1300 containing the GFP (green fluorescent protein) tag was injected into *Nicotiana benthamiana* leaves for subcellular localization using Agrobacterium-mediated transformation. After infecting tobacco with the 35S::GFP vector, green fluorescence can be seen in the whole cell. Green fluorescence in tobacco injected with the 35S::PfMYB90-GFP vector was consistent with red NLS-mCherry fluorescence, indicating that PfMYB90 localizes in the nucleus (Figure 2).

### 2.3. Analysis of Expression Characteristics of PfMYB90

**Figure 3:**
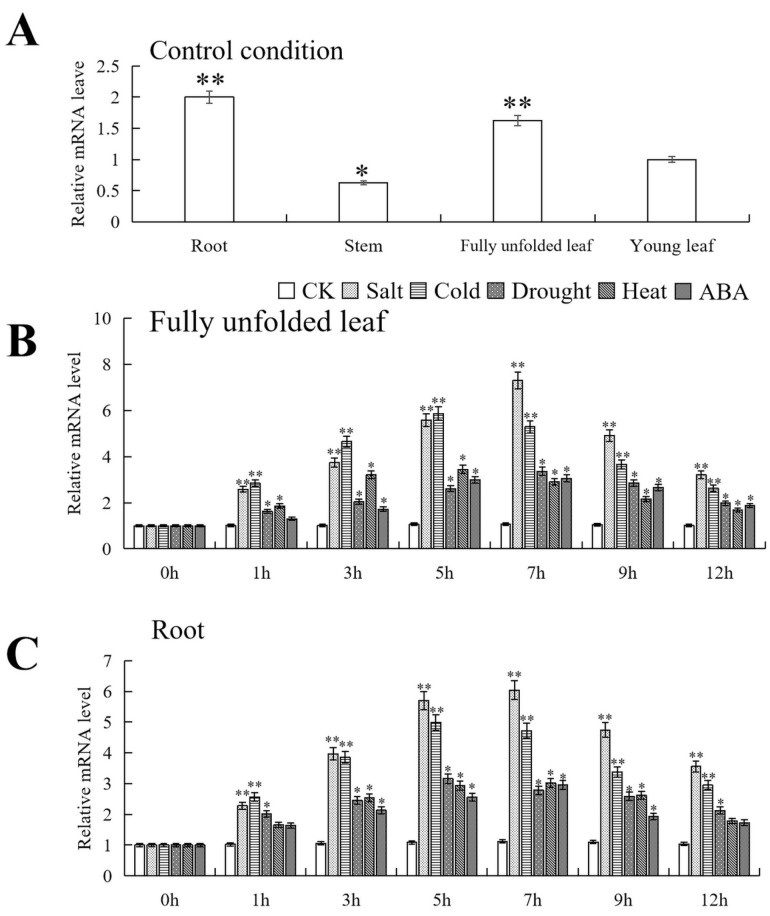
Expression of *PfMYB90*. (**A**) *PfMYB90* expression in various organs. *PfMYB90* expression levels in young leaf as a reference. (**B**) Relative expression of *PfMYB90* under stresses in fully unfolded leaf and (**C**) root. The expression level of 0 h was set to 1 as the control (CK). An error bar (*n* = 3) represents the standard deviation. ** *p*-value ≤ 0.01 and * *p*-value ≤ 0.05.

Using RT-qPCR, we investigated the specific expression of *PfMYB90* in the root, stem, fully unfolded leaf, and young leaf of *Paulownia fortunei*. Compared with the expression level of the young leaf, the expression level of the *PfMYB90* gene in the root was the highest, about 2.0 times that of the young leaf. Next was the fully unfolded leaf, which was about 1.63 times. The expression level in the stem was significantly lower than that in the young leaf, at about 0.63 times (Figure 3A).

By RT-qPCR analysis, *PfMYB90* was found to respond to salt, drought, cold, heat, and ABA stresses. Within 12 h after stress treatments, we observed a unimodal pattern of changes in expression levels. Under salt and ABA stresses, the expression peak of *PfMYB90* in the fully unfolded leaf and root appeared at 7 h after stresses. Under drought stress, the expression of *PfMYB90* in the fully unfolded leaf and root reached its peak at 7 h and 5 h, respectively. Under cold stress, the expression of *PfMYB90* in the fully unfolded leaf and root reached its peak at 5 h. Under heat stress, the peak expression of *PfMYB90* in the fully unfolded leaf and root occurred at 5 h and 7 h after stress, respectively (Figure 3B,C).

### 2.4. Heterologous Expression of PfMYB90 in Arabidopsis Improved Salinity Tolerance

**Figure 4:**
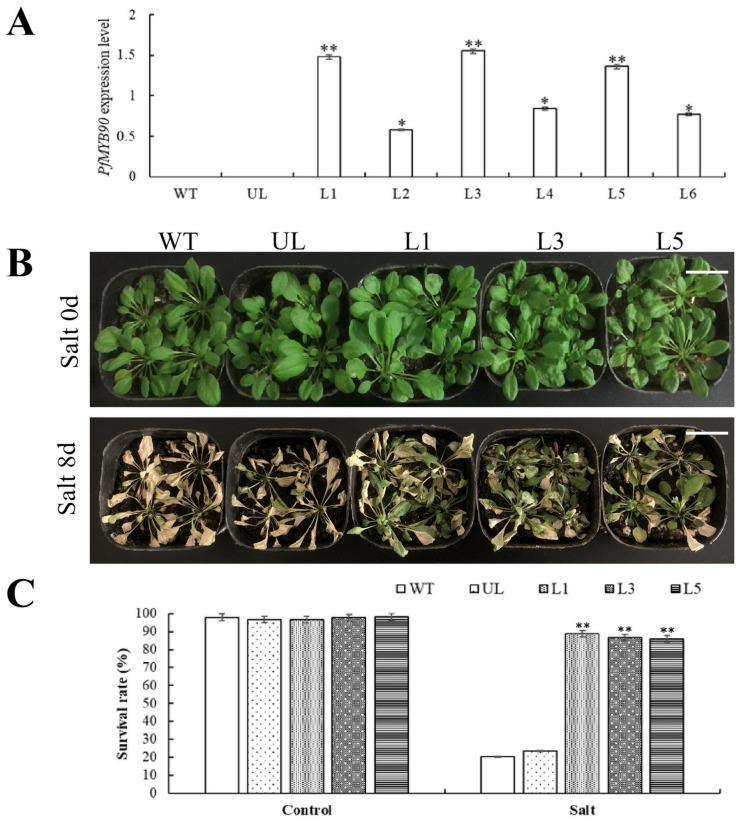
Transgenic-*PfMYB90* enhanced the salt tolerance of *Arabidopsis*. (**A**) *PfMYB90* expression in transgenic lines (L1-6), unloaded lines (UL), and wild type (WT) *Arabidopsis* plants. (**B**) Phenotypes of *Arabidopsis* WT, UL, L1, L3, and L5 after 0 d (Salt 0 d) and 8 d (Salt 8 d) of treatment with 100 mM NaCl. Bar is equal to 4 cm. (**C**) *Arabidopsis* survival rates at Salt 0 d and Salt 8 d. Applying WT as a guide. An error bar (*n* = 3) represents the standard deviation. ** *p*-value ≤ 0.01 and * *p*-value ≤ 0.05.

In order to further investigate the potential regulatory functions of *PfMYB90* under cold and salt stresses, we successfully constructed the *PfMYB90*-pCAMBIA1300 fusion vector. Subsequently, the fusion vector and the original PCAMBIA1300 vector were introduced into *Arabidopsis* by gene transformation technology to cultivate transgenic plants. Using kanamycin screening, we identified positive plants and extracted RNA from them for RT-qPCR analysis. The experimental results showed that *PfMYB90* was not expressed in WT and UL plants but was expressed to varying degrees in positive plants (L1, L2, L3, L4, L5, L6), resulting in transgenic *Arabidopsis*. Three lines with high *PfMYB90* expression (L1, L3, L5) were selected for planting, and their leaves were used for subsequent experiments 20 days later (Figure 4A).

We treated WT, UL, and these three high-expression lines (L1, L3, and L5) with 200 mM NaCl to test the resistance of *PfMYB90* to salt stress. The effects of salt stress on the growth and development of *Arabidopsis* were also observed. At Salt 0 d after the onset of salt stress, the growth status of *Arabidopsis* in the control group (WT, UL) and transgenic-*PfMYB90* group (L1, L3, L5) was similar. However, at Salt 8 d of salt treatment, leaves of both groups showed different degrees of yellowing, but transgenic-*PfMYB90 Arabidopsis* (L1, L3, L5) showed less yellowing, while control plants (WT, UL) showed more severe yellowing (Figure 4B). The survival rate of the plants was calculated. We observed that transgenic-*PfMYB90 Arabidopsis* (L1, 88.8%; L3, 86.7%; and L5, 85.9%) had a higher survival rate than the control group (WT, 20.4% and UL, 23.5%) (Figure 4C). This significant difference further validated the positive role of *PfMYB90* in improving *Arabidopsis* salt-stress resistance.

**Figure 5:**
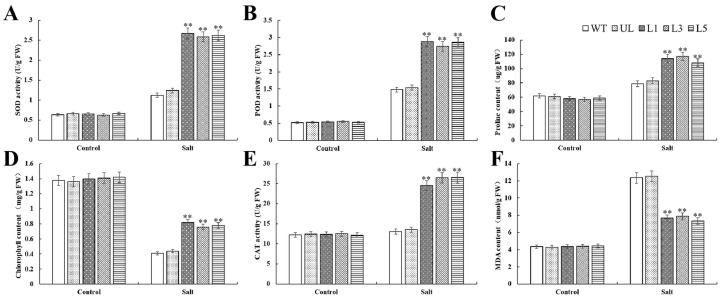
Analysis of (**A**) SOD, (**B**) POD, (**C**) proline, (**D**) chlorophyll, (**E**) CAT, (**F**) and MDA in *Arabidopsis* at 0 d (Salt 0 d) and 8 d (Salt 8 d) of treatment with 100 mM NaCl. Using WT indicators as controls. The SD is represented by an error bar (*n* = 3). ** *p*-value ≤ 0.01.

After salt stress, SOD, POD, and CAT activities and proline and MDA contents of all lines increased (Figure 5A–C,E,F), while chlorophyll contents decreased (Figure 5D). The MDA content of transgenic *Arabidopsis* was significantly lower than that of WT and UL plants (Figure 5D), while the other indexes were just the opposite (Figure 5A–C,E,F).

### 2.5. PfMYB90 Activates Salt-Tolerant Genes in Transgenic Arabidopsis

**Figure 6:**
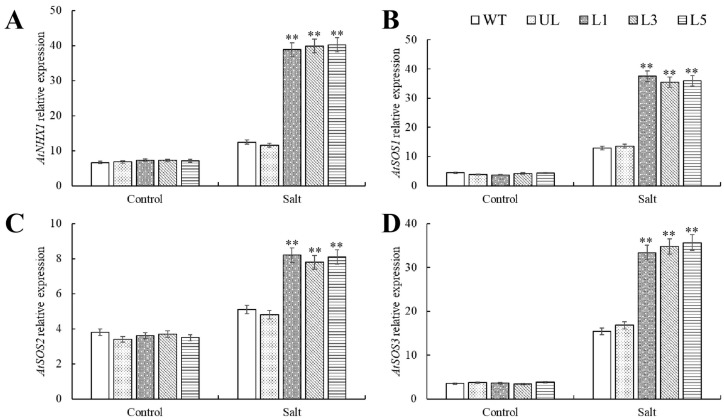
*Arabidopsis* salt-tolerance-related gene expression was detected by RT-qPCR. Relative expression levels of (**A**) *AtNHX1*, (**B**) *AtSOS1*, (**C**) *AtSOS2*, and (**D**) *AtSOS3* after 0 d (Salt 0 d) and 8 d (Salt 8 d) of treatment with 100 mM NaCl. Using WT as control. The SD is represented by an error bar (*n* = 3). ** *p*-value ≤ 0.01.

The *AtNHX1*, *AtSOS1*, *AtSOS2*, and *AtSOS3* gene expression levels were evaluated using RT-qPCR. The results showed that in every *Arabidopsis* plant that did not receive stress treatment, there were no appreciable variations in the expression levels of these genes. However, salt stress treatment enhanced the expression levels of the aforementioned genes in *Arabidopsis*, especially in transgenic-*PfMYB90 Arabidopsis*. It can be inferred that *PfMYB90* may relieve osmotic pressure by activating the expression of salt stress response genes, maintain the water potential of cells, prevent water loss caused by excessive sodium content, and improve the resistance of plants to salt stress (Figure 6).

### 2.6. Heterologous Expression of PfMYBYB90 in Arabidopsis Improved Cold Tolerance

For the selected WT, UL, and transgenic *Arabidopsis* with good growth, after Cold 0 h, the morphology and growth potential of transgenic-*PfMYB90 Arabidopsis* were not significantly different from those of control WT and UL. After Cold 12 h at −4 °C, the leaf color of WT, UL, L1, L3, and L5 was lightened, and the leaves were thinner and wilted. However, the effects of chilling injury in transgenic-*PfMYB90 Arabidopsis* were less than those in the control group.

**Figure 7:**
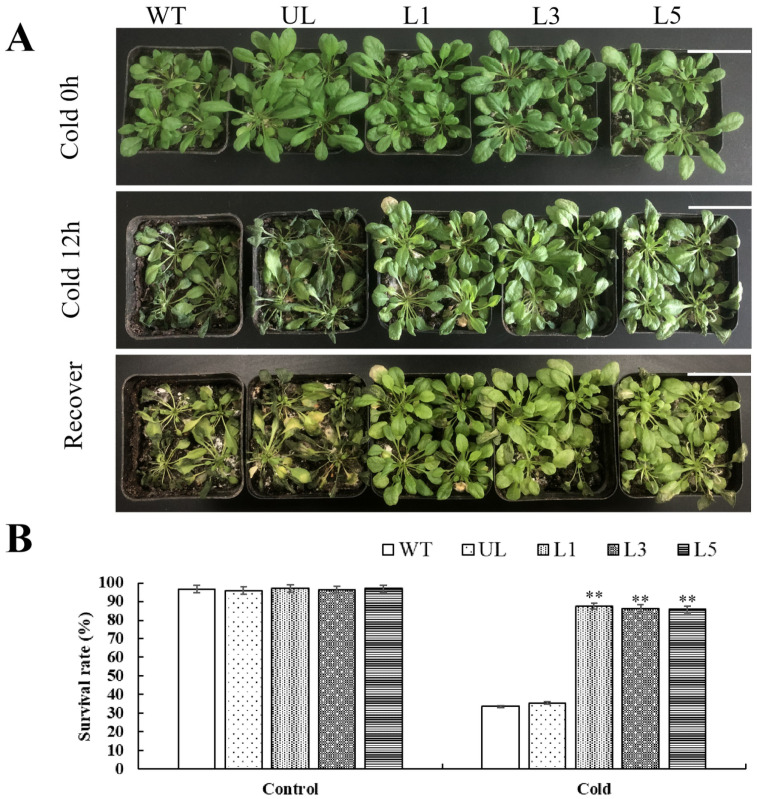
*Arabidopsis* phenotypic alterations and survival rate under cold stress. (**A**) The *Arabidopsis* phenotype (Cold 0 h, 12 h, and recover), with a scale of 4 cm. (**B**) Survival rate of *Arabidopsis* under CK (Cold 0 h) and cold stress (Cold 12 h). The SD is represented by an error bar (*n* = 3). ** *p*-value ≤ 0.01.

After returning to normal conditions for 3 d (recover), it was found that the transgenic-*PfMYB90 Arabidopsis* quickly returned to normal growth. In the control group, WT and UL recovered slowly, and some leaves even died (Figure 7A). Statistics on the survival rate of *Arabidopsis* after growth recovery showed that the survival rate of transgenic-*PfMYB90 Arabidopsis* was significantly higher than that of control group (Figure 7B).

**Figure 8:**
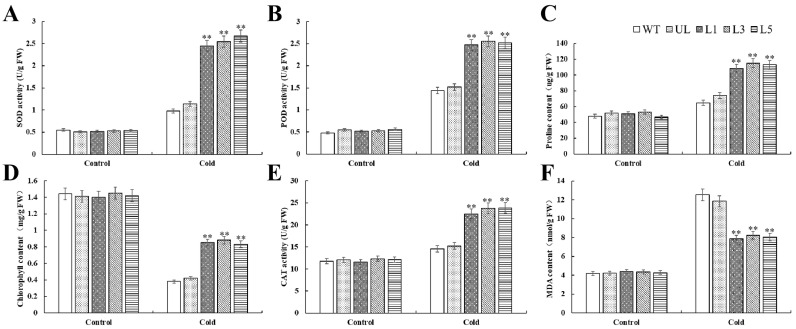
Impact of the *PfMYB90* gene on the *Arabidopsis* cold tolerance index. WT was utilized as the control in (**A**) SOD, (**B**) POD, (**C**) proline, (**D**) chlorophyll, (**E**) CAT, and (**F**) MDA. There is an error bar (*n* = 3) that shows the SD. ** *p*-value ≤ 0.01.

Before cold stress treatment, the physiological indexes of all lines were basically the same, while after cold stress treatment, SOD, POD, and CAT activities and proline and MDA contents were increased, while chlorophyll contents were decreased. In addition, the MDA content of transgenic *Arabidopsis* was significantly lower than that of WT and UL plants, while other indicators were just the opposite (Figure 8).

### 2.7. PfMYB90 Activates Cold-Tolerant Genes in Transgenic Arabidopsis

**Figure 9:**
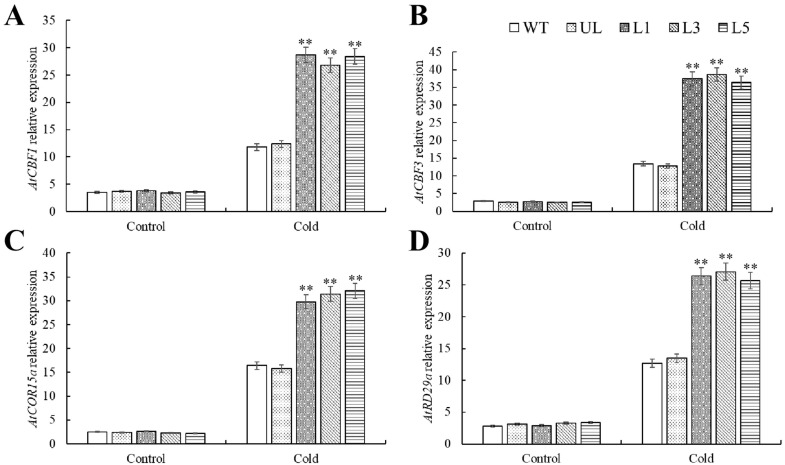
Expression levels of genes linked to cold stress in transgenic, UL, and WT *Arabidopsis* under cold stress conditions. (**A**) *AtCBF1*, (**B**) *AtCBF3*, (**C**) *AtCOR15a*, (**D**) *AtRD29a* relative expression levels. There is an error bar (*n* = 3) that shows the SD. ** *p*-value ≤ 0.01.

We performed RT-qPCR analysis on the signature genes *AtCBF1*, *AtCBF3*, *AtCOR15a,* and *AtRD29a* of cold stress and found that the expression levels of these genes were basically the same in all *Arabidopsis* plants before cold stress treatment, and their expression levels were very low. After 12 h of cold stress, WT, UL, L1, L3, and L5 all showed increases in the expression levels of the cold stress marker genes *AtCBF1*, *AtCBF3*, *AtCOR15a,* and *AtRD29a*. Notably, the expression levels of these genes in transgenic-*PfMYB90* plants were much greater than those in control plants (Figure 9).

## 3. Discussion

Paulownia is frequently used as raw material in traditional Chinese medicine and various industrial applications, highlighting its overall value. MYB TF is the largest family of transcription factors in plants and is widely involved in various physiological activities during plant growth and development. Apart from its noteworthy influence on secondary metabolism [20,21,22,23], MYB TF also plays a role in the development of plant resistance to abiotic stressors [24,25,26]. Therefore, it is particularly necessary to further explore the function of MYB TF family in paulownia, which will greatly promote molecular breeding of paulownia and provide strong scientific support for its genetic improvement. In this study, the *PfMYB90* gene was cloned by homologous cloning of MYB90 (XP_011100106.1) gene in transcriptome sequencing.

*PfMYB90* contains two SANT-MYB domains, which are typical of the R2R3MYB transcription factor protein, and it has the same conserved domain as the MYB90 protein of other species (Figure 1). In this study, we successfully constructed 35S::*PfMYB90*-GFP vector and transferred it into tobacco cells. Through careful observation, we found that the 35S::*PfMYB90*-GFP vector is also located in the nucleus (Figure 2), which is consistent with previous studies, and further confirmed that PfMYB90 has the typical characteristics of TF [27]. We therefore infer that PfMYB90 is a potential transcription factor.

The MYB TF family plays a wide and important role in the mechanisms of plant tolerance to various adverse environments. These transcription factors can effectively regulate a series of physiological and metabolic processes in plants, thereby enhancing their resistance and adaptability to adverse environments. For example, *CsMYB30* validated in citrus altered the degree of wax covering on plant body surfaces and altered the microstructure of wax crystals on stem surfaces to improve the plant’s saline-alkali tolerance [28]. Heterologous up-regulated expression of *FtMYB9* in buckwheat tartare activated key downstream genes of the *Arabidopsis* ABA signaling pathway and improved the saline-alkali resistance of plants [29]. The overexpression of *ZfMYB-IF35* in the model plant *Arabidopsis* improved its ability to tolerate cold stress [30]. The down-regulated expression of *MeMYB2* in sweet potato promotes the synthesis of anthocyanins, thereby indirectly improving the resistance of cold resistant sweet potato to cold injury [31].

In this study, the expression levels of *PfMYB90* in different tissues of paulownia were detected in detail by RT-qPCR technology, so as to explore its potential role in the growth and development and physiological process of paulownia. It was found that the expression level of *PfMYB90* in different parts of the plant was different, and the expression level of the root system was relatively the highest among the tested plant parts (Figure 3A). In addition, we found that, although the expression patterns differed, *PfMYB90* was expressed in fully unfolded leaf and root whether challenged by ABA, heat, cold, drought, and salt stresses. *PfMYB90* responded more strongly to salt and cold stresses, reaching the highest expression levels at 7 h and 5 h, respectively, then decreasing. The expression of *PfMYB90* under drought, heat, and ABA stresses showed the same trend as that under salt and cold stress, but the expression level of *PfMYB90* under these three stresses was relatively low (Figure 3B,C). Therefore, we chose salt and cold stresses as subsequent experimental means to further verify the gene function of *PfMYB90*.

By examining the phenotypic traits of *Arabidopsis*, we discovered that transgenic plants exhibited considerably less damage under salt and cold stressors than the control group, and that their rate of recovery increased following stress alleviation. (Figure 4 and Figure 7). When salt stress and cold stresses are present, the balance of oxygen metabolism in plants will be disturbed. In this state, the production rate of ROS is significantly increased, while the efficiency of the clearance system is decreased, resulting in the gradual accumulation of ROS in the body [32]. This stress state caused by ROS accumulation is called oxidative stress, and the cellular damage caused by it is called oxidative damage [33]. In order to resist physiological damage caused by stresses, plants will initiate a series of complex antioxidant mechanisms and generate a variety of antioxidant enzymes, such as SOD, POD, CAT, APX, and GR. The antioxidant system composed of these enzymes effectively keeps ROS within the limits that cells can tolerate. Our experimental validation revealed that transgenic-*PfMYB90 Arabidopsis* plants markedly improved ROS scavenging capability, as evidenced by markedly elevated activities of POD, SOD, and CAT enzymes (Figure 5 and Figure 8). This finding not only helps to reduce the damage of ROS to plant cell membranes but also effectively reduces the osmotic pressure of cell membranes, thus significantly improving the plant’s ability to tolerate salt and cold stresses.

Malondialdehyde, as the final decomposition product of membrane peroxidation, can indirectly reflect the damage degree of membrane to a certain extent [8]. The permeability of plasma membrane and the content of MDA increased, which aggravated the damage of plasma membrane [34]. In order to mitigate the damage caused by various stresses, plants adopt a mechanism that regulates osmotic pressure within the cell by accumulating specific solutes. In this process, proline plays a central role and becomes a highly efficient osmotic regulator [35]. Proline not only helps to maintain the balance of osmotic pressure inside and outside cells, but its importance is also embodied in many aspects: it can stabilize the structure of biological macromolecules, reduce the acidic environment of cells, relieve the toxicity of ammonia, and also act as an energy bank to regulate the REDOX potential of cells [36]. These combined effects make proline one of the important coping strategies for plants in the face of stress such as salt stress and cold damage. When exposed to salt and cold stress, transgenic-*PfMYB90 Arabidopsis* showed noticeably greater levels of proline and chlorophyll content than the control group, along with significantly higher activity of antioxidant enzymes like SOD, POD, and CAT. Regarding MDA content, the converse was true (Figure 5 and Figure 8). We investigated the difference of tolerance between transgenic-*PfMYB90 Arabidopsis* and its control lines under stress at the molecular level. Special attention was paid to the SOS pathway, which is a key defense mechanism in plants responding to Na^+^ stress [37]. Under the activation of SOS3, SOS2 needs to transfer to the cell membrane, interact with the Na^+^/H^+^ antiporter SOS1 located on the cell membrane, and activate SOS1 through phosphorylation, so as to effectively pump excessive intracellular Na^+^ out of the cell and maintain the intracellular K^+^/Na^+^ balance. And then it must regulate the adaptability of plants to salt stress [38]. The AtSOS3-ATSOS2 complex phosphorylates the AtSOS1 protein, which in turn stimulates the Na^+^/H^+^ exchange that AtSOS1 regulates. AtSOS3 interacts with AtSOS2 protein kinase to boost its activity [39,40]. We additionally concentrated on the function of Na^+^/H^+^ antiporter (NHX1) in addition to the SOS pathway. The vacuole membrane contains the Na^+^/H^+^ reverse transporter, which is encoded by NHX1. It can transfer excess Na^+^ from the cell to the vacuole, preventing Na^+^ from accumulating in the cytoplasm and improving plant salt tolerance [41,42]. Our findings demonstrated that the expression of genes linked to SOS and NHX1 was positively regulated by salt stress. In transgenic-*PfMYB90 Arabidopsis*, the expression levels of *AtSOS1/2/3* and *AtNHX1* were considerably higher than in the control group. This study discovered that *PfMYB90* greatly increased the salt tolerance of plants by favorably regulating the expression of *AtSOS1/2/3* and *AtNHX1*, which maintained the balance of intracellular Na^+^ content (Figure 6). Research has demonstrated that MYB TF can bind selectively to the CBF promoter region, offering a fresh viewpoint on how plants regulate their genes in cold environments [43,44]. Specifically, CBF proteins play a crucial role in plants by interacting with CRT/DRE cis-acting elements to trigger the expression of a series of downstream genes such as CORs, RDs, and LTIs, the expression of which is crucial for the improvement of cold tolerance in plants [45]. We particularly investigated two core CBF pathway genes, CBF1 and CBF3, as well as two important cold response genes that are downstream of them, COR15a and RD29a, in order to better investigate the gene regulatory mechanisms in the CBF system. According to the results, *PfMYB90* could significantly increase the expression of *AtCBF1*, *AtCBF3*, *AtCOR15a*, and *AtRD29a* in transgenic *Arabidopsis* under cold stress (Figure 9). This finding not only provides a new perspective for understanding the role of MYB TFs in plant stress resistance but also suggests that *PfMYB90* can actively participate in plant response to cold stress through CBF-dependent pathways, thereby enhancing plant cold tolerance.

**Figure 10:**
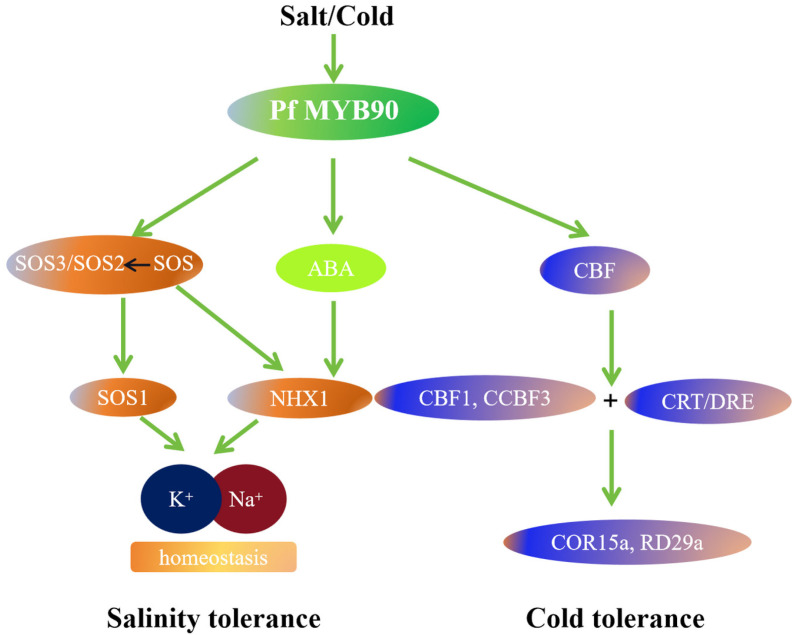
A potential mechanistic model of PfMYB90 adaptation to cold and salt stress.

To put it briefly, we developed a hypothetical regulatory network model focused on *PfMYB90* based on the results of the experiment mentioned above (Figure 10). The SOS pathway was activated under salt stress due to a significant upregulation of *PfMYB90* expression. Na^+^ is transported and excreted by *AtSOS1* in cells, and the SOS3-SOS2 kinase complex controls *AtSOS1* activity. Furthermore, the SOS pathway and ABA can regulate the expression of *AtNHX1* and maintain the intracellular K^+^/Na^+^ balance, both of which improve plants’ resistance to salt. Following the induction of *PfMYB90* expression by cold stress, *PfMYB90* binds to the CBF promoter region. Two important genes in the CBF-dependent pathway, CBF1 and CBF3, then bind to the CRT/DRE cis-acting elements of the downstream cold response genes, COR15a and RD29a, directly activating their expression and resulting in cold resistance. In summary, overexpression of *PfMYB90* is beneficial to the ability of plants to resist cold and salt stresses.

## 4. Materials and Methods

### 4.1. Plant Materials, Growing Conditions, and Handling

In the 322 laboratory of the Northeast Agricultural University’s College of Horticulture and Landscape Architecture, paulownia seedlings were grown as experimental materials in a tissue culture room (humidity: 75%, 25 °C, light: 16 h, darkness: 8 h). Paulownia hydroponic seedlings (about 5–6 cm in height) treated with ABA, salt, cold, drought, and heat prior to the experiment had comparable growth. The hydroponic seedlings were placed in Hoagland solution for salt (Hoagland solution containing 100 mM NaCl, Coolaber, Beijing, China), cold (4 °C Hoagland solution), drought (Hoagland solution containing 20% PEG6000, Coolaber, Beijing, China), heat (37 °C Hoagland solution), and ABA (Hoagland solution containing 100 μM ABA, Coolaber, Beijing, China) treatments. RT-qPCR was used to examine the expression profile of *PfMYB90* during stress periods of 0, 1, 3, 5, 7, 9, and 12 h. *Arabidopsis* grew in a light incubator with 75% humidity, 25 °C, 16 h of light, and 8 h of darkness.

### 4.2. PfMYB90 Cloning and Bioinformatic Analysis

After the extraction of total RNA from paulownia leaves (MolPure^®^ Plant RNA Kit, Laguna Hills, CA, USA), the first cDNA was reverse-transcribed with the TransScript^®^ One-Step gDNA Removal and cDNA Synthesis Super Mix (TransGen Biotech, Beijing, China). Cloning primers *PfMYB90*-F: ATGGAAAAGAATCGAGTAGGAGTG and *PfMYB90*-R: CTAATTGCAGTGCATGAATTGG of the target gene *PfMYB90* were designed according to the sequence of MYB90 (XP_011100106.1) in *Paulownia fortunei* transcriptome data. Primers are ordered from 5′ to 3′. The PCR amplification product of the target gene was linked to the T_5_ cloning vector. The positive single colonies obtained by kanamycin screening were sequenced.

Homology analysis of PfMYB90 protein was performed using NCBI (https://www.ncbi.nlm.nih.gov/, accessed on 23 January 2022) and DNAMAN5.2. MEGA11 was used to construct an evolutionary tree and analyze the evolutionary relationship of PfMYB90. The physicochemical properties of PfMYB90 were analyzed using TBtools v2.042. The secondary and tertiary structures of PfMYB90 were analyzed using SOPMA (https://npsa-prabi.ibcp.fr/, accessed on 26 February 2022) and SWISS-MODEL (https://swissmodel.expasy.org/, accessed on 26 February 2022).

### 4.3. Vector Construction and Subcellular Localization of PfMYB90

Linearization of the pCAMBIA1300-GFP vector was performed using restriction endonucleases *BamH*I and *Sal*I [46]. We added 15 bp homologous arms at both ends of the cloned primers and used homologous recombination to connect the target gene with a linearized vector to construct the fusion vector *PfMYB90*-pCAMBIA1300 [47]. To determine the localization of PfMYB90 in cells, Agrobacterium GV3101 containing *PfMYB90*-pCAMBIA1300 vector and pCAMBIA1300-GFP vector were injected into NLS-mCherry transgenic tobacco leaves cultured for 30 d [48], and fluorescence localization was observed.

### 4.4. Expression Analysis of PfMYB90

We used specific primers PfMYB90-qF/qR: 5′-ACAGATGCAGGAAGAGTTG-3′/5′- TGTGAAGCC TTACAATGAGA-3′ to analyze the expression levels of key transcription factors *PfMYB90* in response to abiotic stress. *PfActin* (F-5′-AATGGAATCTGCTGGAAT-3′/R-5′-ACTGAGGACAATGTTACC-3′) was selected as the internal reference gene. The HiScript QRT SuperMix for qPCR (+gDNA wiper) (R123) kit (Vazyme, Nanjing, China) was used. In the specific experiment, two-step RT-qPCR detection was carried out. The detailed methodology draws on the work of Han et al. [49], which mainly included pre-denatured (95 °C, 30 s) and a 40-cycle circulatory system (95 °C, 10 s; 60 °C, 30 s). 2^−∆∆Ct^ calculated the relative expression of *PfMYB90* [49].

### 4.5. Generation of Transgenic Arabidopsis

Agrobacterium containing pCAMBIA1300 vector and *PfMYB90*-pCAMBIA1300 vector was activated by secondary activation [50]. When the Agrobacterium solution OD600 = 1, the re-suspension solution (OD600 = 0.5–0.8) was prepared. Flowering Columbia ecotype wild-type (WT) *Arabidopsis* was immersed in re-suspension solution for 30 s, then left to rest for 5 min and was cultured in the dark for 24 h. In order to improve the success rate of *Arabidopsis* transformation, each batch of seedlings was infected 2–3 times, with an interval of 1 week.

The T_1_ generation of *Arabidopsis* was the first seedlings obtained by screening infected seeds with kanamycin (50 mg L^−1^). The screened seedlings were then planted until T_2_ seeds were harvested. After seeding T_2_ generation *Arabidopsis*, in order to obtain homozygous T_3_ *Arabidopsis*, RT-qPCR was used to detect the expression of *PfMYB90* in T_2_ generation *Arabidopsis*, and high expression lines (the expression of WT was compared) were further cultivated. Then, *Arabidopsis* T_3_ was treated with cold and salt stresses [51].

### 4.6. Examination of Associated Physiological Indices in Arabidopsis

We selected wild-type (WT, plants that had not been infected by Agrobacterium) and unloaded line (UL, plants infected with Agrobacterium tumefaciens containing pCAMBIA1300 vector) plants and transgenic *Arabidopsis* (L1, L3, and L5) with uniform growth, and we measured the corresponding physiological indexes under different stress conditions. For each *Arabidopsis* line, we divided it into three groups, one of which was grown under normal conditions, while the other two groups were treated under salt stress and cold stress, respectively. Specifically, the salt stress treatment was achieved by irrigating Hoagland solution containing 200 mM NaCl for 8 consecutive days, while the cold damage stress lasted for 12 h at −4 °C. The survival rate of *Arabidopsis* was calculated after salt stress treatment. The growth of *Arabidopsis* under cold stress was resumed and after three days, and the survival rate was determined. Next, we assessed the corresponding physiological indices and closely examined the phenotypic alterations of the plants both before and after stress treatment. These indicators include chlorophyll content (determined by anhydrous ethanol extraction) [52], proline content (extracted with sulfonic salicylic acid and chromogenic reaction with indanhydrin) [53], and malondialdehyde content (extracted with thiobarbituric acid) [54], as well as the activities of peroxidase (POD), superoxide dismutase (SOD), and catalase (CAT) (determined by the test kit of Suzhou Grace Biotechnology Co., Ltd., Suzhou, China, project number: 890).

### 4.7. Stress-Related Gene Expression Analysis in Arabidopsis

Under salt stress (treated with 200 mM NaCl for 8 d) and cold stress (treated at −4 °C for 12 h), the RNA of *Arabidopsis* (WT, UL, L1, L3, L5) was extracted, and cDNA was obtained by reverse transcription. The expressions of downstream genes *AtNHX1*, *AtSOS1*, *AtSOS2*, *AtSOS3*, *AtCBF1*, *AtCBF3*, *AtCOR15a,* and *AtRD29a* were detected by RT-qPCR. *AtActin* was selected as the internal reference gene. Specific primers are as follows: AtNHX1-F/R: AGCCTTCAGGGAACCACAAT/CT CCAAAGACGGGTCGCATG; AtSOS1-F/R: TTCATCATCCTCACAATGGCTCTAA/CCCTCATCA AGCATCTCCCAGTA; AtSOS2-F/R: GCAAGGGAAGAAGAAGAAGT/TCTCCGCTACATAACTG CC; AtSOS3-F/R: GAATCCATCGCTCATCAA/CCATTTCTTCCTCTTCACA; AtCBF1-F/R: TCGGGACTTTCCAAACCG/CCATCTCCTTCGCCGTCAT; AtCBF3-F/R: TCCGGTAAGTGGG TTTGTGAG/AACTCGGCATCTCAAACATCG; AtCOR15a-F/R: CAACAGAGGAATCACCAGC GA/CTCTGCTGTCTTGTCGTGGTGT; AtRD29a-F/R: CAACGAGGGGAAGATAAAAGTGT/AGCCAGATGATTTTGGAGCCT. The primer sequence is from 5′ to 3′.

### 4.8. Statistical Analysis

The physiological index data of *Arabidopsis* lines (WT, UL, L1, 3, 5) before and after stress were analyzed using the GraphPad principle (v8.0.2.263) software, and the standard error (±SE) and difference (* *p* ≤ 0.05, ** *p* ≤ 0.01) were labeled. Three repetitions of the biological and technological phases were carried out.

## 5. Conclusions

PfMYB90 is an R2R3-type MYB transcription factor (TF) identified by paulownia transcriptome sequencing. Under salt and cold stresses, *PfMYB90* expression levels in paulownia roots and fully unfolded leaf increased significantly. In order to further investigate the function of *PfMYB90*, overexpression-*PfMYB90 Arabidopsis* was constructed, and their phenotypes and stress-related physiological indicators were analyzed in detail. As a result of the allogeneic expression of *PfMYB90*, *Arabidopsis thaliana* was able to respond to salt stress and cold temperatures much better. Furthermore, we further analyzed the expression levels of genes associated with stress response downstream of MYB, which also supported the above findings. The in-depth study on the function of *PfMYB90* not only provides clues for understanding the specific role of *PfMYB90* in paulownia’s response to environmental stress but also provides new possibilities and ideas for the molecular breeding of paulownia.

## Data Availability

The original data for this present study are available from the corresponding authors.

## Supplementary Materials

The following supporting information can be downloaded at: https://www.mdpi.com/article/10.3390/plants14010024/s1, Figure S1: Nucleotide and amino acid sequence of *PfMYB90*. The yellow and green underlined part was the R-repeated conserved domain; Figure S2: Structure of the PfMYB90 protein. (A) secondary structure, (B) functional domain, and (C) tertiary structure of PfMYB90.

## References

[1] Lu X., Liu Y., Xu J., Liu X., Chi Y., Li R., Mo L., Shi L., Liang S., Yu W. (2023). Recent progress of molecular mechanisms of DNA methylation in plant response to abiotic stress. Environ. Exp. Bot..

[2] Ng D.W.K., Abeysinghe J.K., Kamali M. (2018). Regulating the regulators: The control of transcription factors in plant defense signaling. Int. J. Mol. Sci..

[3] Xu Z., Wang T., Hou S., Ma J., Li D., Chen S., Gao X., Zhao Y., He Y., Yang G. (2024). A R2R3-MYB, *BpMYB1*, from paper mulberry interacts with DELLA protein BpGAI1 in soil cadmium phytoremediation. J. Hazard..

[4] Abubakar A., Feng X., Gao G., Yu C., Chen J., Chen K., Wang X., Mou P., Shao D., Chen P. (2022). Genome wide characterization of R2R3 MYB transcription factor from *Apocynum venetum* revealed potential stress tolerance and flavonoid biosynthesis genes. Genomics.

[5] Zhou L., Yarra R., Jin L., Cao H. (2020). Genome-wide identification and expression analysis of MYB gene family in oil palm (*Elaeis guineensis* Jacq.) under abiotic stress conditions. Environ. Exp. Bot..

[6] Leng B., Wang X., Yuan F., Zhang H., Lu C., Chen M., Wang B. (2021). Heterologous expression of the *Limonium bicolor* MYB transcription factor *LbTRY* in *Arabidopsis thaliana* increases salt sensitivity by modifying root hair development and osmotic homeostasis. Plant Sci..

[7] Baldoni E., Genga A., Cominelli E. (2015). Plant MYB transcription factors: Their role in drought response mechanisms. Int. J. Mol. Sci..

[8] So K., Wang J., Sun S., Che H., Zhang Y. (2024). Comprehensive analysis of MYB gene family and their expression under various stress conditions in *Lilium pumilum*. Sci. Hortic..

[9] Li C., Ng C.K.Y., Fan L.M. (2015). MYB transcription factors, active players in abiotic stress signaling. Environ. Exp. Bot..

[10] Li J., Zhou H., Xiong C., Peng Z., Du W., Li H., Wang L., Ruan C. (2022). Genome-wide analysis R2R3-MYB transcription factors in *Xanthoceras sorbifolium* Bunge and functional analysis of *XsMYB30* in drought and salt stresses tolerance. Ind. Crop. Prod..

[11] Zhu N., Cheng S., Liu X., Du H., Dai M., Zhou D., Yang W., Zhao Y. (2015). The R2R3-type MYB gene *OsMYB91* has a function in coordinating plant growth and salt stress tolerance in rice. Plant Sci..

[12] Zhao Y., Yang Z., Ding Y., Liu L., Han X., Zhan J., Wei X., Diao Y., Qin W., Wang P. (2019). Over-expression of an R2R3 MYB Gene, *GhMYB73*, increases tolerance to salt stress in transgenic *Arabidopsis*. Plant Sci..

[13] Cao X., Zhai X., Zhao Z., Deng M., Li Y., Fan G. (2020). Genome-wide DNA methylation analysis of paulownia with phytoplasma infection. Gene.

[14] Morote L., Rubio-Moraga A., López-Jiménez A.J., Argandoña J., Niza E., Ahrazem O., Gómez-Gómez L. (2023). A carotenoid cleavage dioxygenase 4 from *Paulownia tomentosa* determines visual and aroma signals in flowers. Plant Sci..

[15] Chen J., Liu Y., Shi Y.P. (2009). Determination of flavonoids in the flowers of *Paulownia tomentosa* by high-performance liquid chromatography. J. Anal. Chem..

[16] Xu E., Fan G., Niu S., Zhao Z., Deng M., Dong Y. (2015). Transcriptome sequencing and comparative analysis of diploid and autotetraploid *Paulownia australis*. Tree Genet. Genomes.

[17] Guo N., Zhai X.Q., Fan G.Q. (2023). Chemical composition, health benefits and future prospects of Paulownia flowers: A review. Food Chem..

[18] Huber C., Moog D., Stingl R., Pramreiter M., Stadlmann A., Baumann G., Praxmarer G., Gutmann R., Eisler H., Müller U. (2023). Paulownia (*Paulownia elongata* S.Y.Hu)—Importance for forestry and a general screening of technological and material properties. Wood Mater. Sci. Eng.

[19] Yao H.Y., Gao Z.Y., Wang J.L. (2016). Research on the biology and medicinal value of *Botrytis cinerea*. J. Weinan Norm. Coll..

[20] Liu J.Y., Osbourn A., Ma P.D. (2015). MYB transcription factors as regulators of phenylpropanoid metabolism in plants. Mol. Plant.

[21] Zhang Y., Hu J., Li L., Zhang X., Chen L., Zhou Z., Wang J., Sheng Q., Liang Z., Hong G. (2024). Single-repeat MYB transcription factor, *OsMYB1R*, enhanced phytoalexin sakuranetin accumulation and Magnaporthe oryzae resistance. Curr. Opin. Plant Biol..

[22] Xie D.W., Li J., Zhang X.Y., Dai Z.G., Zhou W.Z., Su J.G., Sun J. (2023). Systematic analysis of MYB transcription factors and the role of *LuMYB216* in regulating anthocyanin biosynthesis in the flowers of flax (*Linum usitatissimum* L.). J. Integr. Agric..

[23] Zheng W., Dong X.M., Zhang Q.Y., Yu X.F., Li W.L., Ji Y.B., Chen N. (2020). Identification and characterization of MYB genes in *Dimocarpus Longan* Lour. Bangl. J. Bot..

[24] Wang X.P., Niu Y.L., Zheng Y. (2021). Multiple functions of MYB transcription factors in abiotic stress responses. Int. J. Mol. Sci..

[25] Zhou Z., Wei X., Lan H. (2023). *CgMYB1*, an R2R3-MYB transcription factor, can alleviate abiotic stress in an annual halophyte *Chenopodium glaucum*. Plant Physiol. Biochem..

[26] Wang Y., Wu J., Li J., Liu B., Wang D., Gao C. (2023). The R2R3-MYB transcription factor *ThRAX2* recognized a new element MYB-T (CTTCCA) to enhance cadmium tolerance in *Tamarix hispida*. Plant Sci..

[27] Yu Y., Guo D.D., Min D.H., Cao T., Ning L., Jiang Q.Y., Sun X.J., Zhang H., Tang W.S., Gao S.Q. (2023). Foxtail millet MYB-like transcription factor *SiMYB16* confers salt tolerance in transgenic rice by regulating phenylpropane pathway. Plant Physiol. Biochem..

[28] Wen X.F., Geng F., Cheng Y.J., Wang J.Q. (2021). Ectopic expression of *CsMYB30* from Citrus sinensis enhances salt and drought tolerance by regulating wax synthesis in *Arabidopsis thaliana*. Plant Physiol. Biochem..

[29] Gao F., Zhou J., Deng R.Y., Zhao H.X., Li C.L., Chen H., Suzuki T., Park S.U., Wu Q. (2017). Overexpression of a tartary buckwheat R2R3–MYB transcription factor gene, *FtMYB9*, enhances tolerance to drought and salt stresses in transgenic *Arabidopsis*. J. Plant Physiol..

[30] Meng C., Sui N. (2019). Overexpression of maize *MYB–IF35* increases chilling tolerance in *Arabidopsis*. Plant Physiol. Biochem..

[31] Guo X., Yu X.H., Lin C.Y., Zhao P.J., Wang B., Zou L.P., Li S.X., Yu X.L., Chen Y.H., Zhang P. (2023). Down–regulation of *MeMYB2* leads to anthocyanin accumulation and increases chilling tolerance in cassava (*Manihot esculenta* Crantz). Crop J..

[32] Gill S.S., Tuteja N. (2010). Reactive oxygen species and antioxidant machinery in abiotic stress tolerance in crop plants. Plant Physiol. Biochem..

[33] Hasanuzzaman M., Bhuyan M.H.M.B., Zulfiqar F., Raza A., Mohsin S.M., Al Mahmud J., Fujita M., Fotopoulos V. (2020). Reactive oxygen species and antioxidant defense in plants under abiotic stress: Revisiting the crucial role of a universal defense regulator. Antioxidants.

[34] Liu L., Yuan Y.D., Zuo J.J., Tao J. (2023). Composition and antioxidant activity of *Paeonia lactiflora* petal flavonoid extract and underlying mechanisms of the protective effect on H_2_O_2_–induced oxidative damage in BRL3A cells. Hortic. Plant J..

[35] Ghosh U.K., Islam M.N., Siddiqui M.N., Cao X., Khan M.A.R. (2022). Proline, a multifaceted signalling molecule in plant responses to abiotic stress: Understanding the physiological mechanisms. Plant Biol..

[36] Kishor P.B.K., Kumari P.H., Sunita M.S.L., Sreenivasulu N. (2015). Role of proline in cell wall synthesis and plant development and its implications in plant ontogeny. Front. Plant Sci..

[37] Ali A., Petrov V., Yun D.J., Gechev T. (2023). Revisiting plant salt tolerance: Novel components of the SOS pathway. Trends Plant Sci..

[38] Zhou X.Y., Li J.F., Wang Y.Q., Liang X.Y., Zhang M., Lu M.H., Guo Y., Qin F., Jiang C.F. (2022). The classical SOS pathway confers natural variation of salt tolerance in maize. New Phytol..

[39] Ma L., Liu X., Lv W., Yang Y. (2022). Molecular mechanisms of plant responses to salt stress. Front. Plant Sci..

[40] Quintero F.J., Martinez-Atienza J., Villalta I., Jiang X.Y., Kim W.Y., Ali Z., Fujii H., Mendoza I., Yun D.J., Zhu J.K. (2011). Activation of the plasma membrane Na/H antiporter Salt-Overly-Sensitive 1 (SOS1) by phosphorylation of an auto-inhibitory C-terminal domain. Proc. Natl. Acad. Sci. USA.

[41] Fan Y., Wan S., Jiang Y., Xia Y.Q., Chen X.H., Gao M.Z., Cao Y.X., Luo Y.H., Zhou Y., Jiang X.Y. (2018). Over–expression of a plasmamembrane H^+^-ATPase *SpAHA1* conferred salt tolerance to transgenic *Arabidopsis*. Protoplasma.

[42] Guo Q., Tian X.X., Mao P.C., Meng L. (2020). Overexpression of Iris lactea tonoplast Na^+^/H^+^ antiporter gene *IlNHX* confers improved salt tolerance in tobacco. Biol. Plant..

[43] Agarwal M., Hao Y.J., Kapoor A., Dong C.H., Fujii H., Zheng X.W., Zhu J.K. (2006). A R2R3 type MYB transcription factor is involved in the cold regulation of CBF genes and in acquired freezing tolerance. J. Biol. Chem..

[44] Artlip T.S., Wisniewski M.E., Norelli J.L. (2014). Field evaluation of apple overexpressing a peach CBF gene confirms its effect on cold hardiness, dormancy, and growth. Environ. Exp. Bot..

[45] Li W.H., Zhong J.L., Zhang L.H., Wang Y., Song P.H., Liu W.D., Li X.G., Han D.G. (2022). Overexpression of a *Fragaria vesca* MYB transcription factor gene (*FvMYB82*) increases salt and cold tolerance in *Arabidopsis thaliana*. Int. J. Mol. Sci..

[46] Abbas F., Ke Y.G., Zhou Y.W., Yu R.C., Imran M., Amanullah S., Rothenberg D.O., Wang Q., Wang L., Fan Y.P. (2021). Functional characterization of *Hedychium coronarium* J. Koenig *MYB132* confers the potential role in floral aroma synthesis. Plants.

[47] Wang Z., Mao Y., Guo Y., Gao J., Liu X., Li S., Lin Y.C.J., Chen H., Wang J.P., Chiang V.L. (2020). MYB transcription factor161 mediates feedback regulation of *secondary wall-associated NAC-Domain1* family genes for wood formation. Plant Physiol..

[48] Tang Y.H., Bao X.X., Zhi Y.L., Wu Q., Guo Y.R., Yin X.H., Zeng L.Q., Li J., Zhang J., He W.L. (2019). Overexpression of a MYB family gene, *OsMYB6*, increases drought and salinity stress tolerance in transgenic rice. Front. Plant Sci..

[49] Han J., Li X., Li W., Yao A., Niu C., Hou R., Liu W., Wang W., Zhang L., Han D. (2023). Overexpression of *Malus baccata* WRKY40 (*MbWRKY40*) enhances stress tolerance in *Arabidopsis* subjected to cold and drought. Plant Stress.

[50] Mei G.G., Chen A., Wang Y.R., Li S.Q., Wu M.Y., Hu Y.L., Liu X., Hou X.L. (2024). A simple and efficient in planta transformation method based on the active regeneration capacity of plants. Plant Commun..

[51] Liu X., Sun M., Li X., He R., Qu L., Tong D., Zhao X. (2018). Function analysis on F-box gene FOF2 response to salt stress and cold stress in *Arabidopsis*. J. Hunan Univ. (Nat. Sci.).

[52] Sarkar S., Manna M.S., Bhowmick T.K., Gayen K. (2020). Extraction of chlorophylls and carotenoids from dry and wet biomass of isolated *Chlorella Thermophila*: Optimization of process parameters and modelling by artificial neural network. Process Biochem..

[53] Furlan A.L., Bianucci E., Giordano W., Castro S., Becker D.F. (2020). Proline metabolic dynamics and implications in drought tolerance of peanut plants. Plant Physiol. Biochem..

[54] Zörb C., Geilfus C.M., Dietz K.J. (2019). Salinity and crop yield. Plant Biol..

