# Supplementary material for: Heterologous Expression of a Potential ‘Paulownia fortunei’ MYB Factor Gene, PfMYB90, Improves Salt and Cold Tolerance in Arabidopsis
Source: Plants (Basel). 2024 Dec 25;14(1):24. doi: 10.3390/plants14010024 (PMC11723189; doi:10.3390/plants14010024)
Supplement: Supplementary file 1 [file plants-14-00024-s001.zip › plants-3277596-supplementary.pdf]

```
1   ATGGAAAAGAATCGAGTAGGAGTGAGGAAAGGTGCGTGGACAAAAGAAGAAGATATTCTCTTGAGGAAATGCATTGAAAAGTATGGTGAA
1    M  E  K  N  R  V  G  V  R  K  G  A  W  T  K  E  E  D  I  L  L  R  K  C  I  E  K  Y  G  E
91  GGGAAGTGGCATCTAGTCCCTCTTAGAGCTGGGCTGAACAGATGCAGGAAGAGTTGCAGGCTGAGATGGTTGAACTATCTGAGACCAAAT
31   G  K  W  H  L  V  P  L  R  A  G  L  N  R  C  R  K  S  C  R  L  R  W  L  N  Y  L  R  P  N
181 ATTAAAAGAGGTTACTTTACAAACGATGAAGTGGATCTCATTGTAAGGCTTCACAAGTTGTTAGGAAACAGATGGTCTCTGATTGCTGGT
61   I  K  R  G  Y  F  T  N  D  E  V  D  L  I  V  R  L  H  K  L  L  G  N  R  W  S  L  I  A  G
271 AGACTTCCCGGAAGAACAGGAAACGATGTGAAGAACTTCTGGAACACCCACATTGAGAAGAAGCCAGCAGCTGCTGGAGAAGGTTGCAGG
91   R  L  P  G  R  T  G  N  D  V  K  N  F  W  N  T  H  I  E  K  K  P  A  A  A  G  E  G  C  R
361 GGAAAAACCATTCAAAAAACCATCACTGAGAGCAACATCATAAGACCCCGACCTCGGACCTTCTCCAAATTACAACTACCAGCTTGGCCC
121  G  K  T  I  Q  K  T  I  T  E  S  N  I  I  R  P  R  P  R  T  F  S  K  L  Q  L  P  A  W  P
451 GTTGAACTAACCAAACCAAACGAACCGAATGATGGAAATCCCAAGAACAAGAAGCCATCTTCAACTGCATCATCATCAAAATATTGGAAA
151  V  E  L  T  K  P  N  E  P  N  D  G  N  P  K  N  K  K  P  S  S  T  A  S  S  S  K  Y  W  K
541 ACTGATGATGAGAATCCCAAGAACAAGCAGCCTTCTTCATCTGCATCATTGTCACAAGAAGAGCCAAATGATTGGACAAGTAGCGATGAA
181  T  D  D  E  N  P  K  N  K  Q  P  S  S  S  A  S  L  S  Q  E  E  P  N  D  W  T  S  S  D  E
631 AATCCCAAGAACAAGCAGCCATCTGCATCATCATCAGAAGAAGTAGATGAATGTATTCGGTGGTGGCGCAACTTGTTTGAAATAACTGAA
211  N  P  K  N  K  Q  P  S  A  S  S  S  E  E  V  D  E  C  I  R  W  W  R  N  L  F  E  I  T  E
721 AATGGAGAAGGAACCCCGTTTTCGTTTTCCGACGAGGACCACCGAATTATGGAGCCAATATTATCGCCAGGATTCCATAATCATGCAAAC
241  N  G  E  G  T  P  F  S  F  S  D  E  D  H  R  I  M  E  P  I  L  S  P  G  F  H  N  H  A  N
811 AATTACGTCAAGCAAGATGGATTGAGTAGTTTATCCGTAGATGTTGATGTTTGGGAACTTGTAAGCTTGGACGACCAATTCATGCACTGC
271  N  Y  V  K  Q  D  G  L  S  S  L  S  V  D  V  D  V  W  E  L  V  S  L  D  D  Q  F  M  H  C
901 AATTAG
301  N  *
```

Figure S1. Nucleotide and amino acid sequence of *PfMYB90*. The yellow and green underlined part was the R-repeated conserved domain.

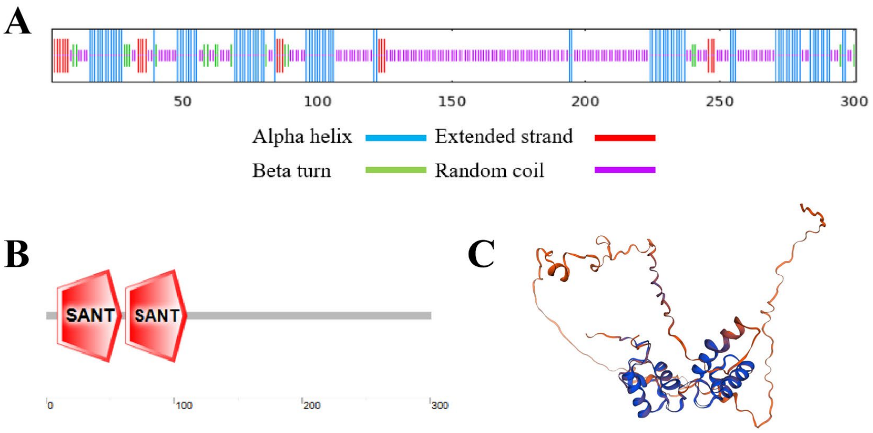

Figure S2. Structure of the PfMYB90 protein. the (**A**) secondary structure, (**B**) functional domain, and (**C**) tertiary structure of PfMYB90.
